# Autoimmune diabetes mellitus after COVID-19 vaccination in adult population: a systematic review of case reports

**DOI:** 10.1186/s12902-023-01424-0

**Published:** 2023-08-04

**Authors:** Ali S Alsudais, Raghad S Alkanani, Abdulaziz B Fathi, Saleh S Almuntashiri, Jafar N Jamjoom, Mustafa A Alzhrani, Alaa Althubaiti, Suhaib Radi

**Affiliations:** 1 College of Medicine, King Saud bin Abdulaziz University for Health Sciences, Jeddah, Saudi Arabia; 2 King Abdullah International Medical Research Centre, Jeddah, Saudi Arabia; 3 Department of Internal Medicine, Division of Endocrinology, Ministry of the National Guard-Health Affairs, Jeddah, Saudi Arabia

**Keywords:** COVID-19 vaccine, Autoimmune diabetes mellitus, Type 1 diabetes mellitus

## Abstract

**Background:**

Autoimmune/type 1 diabetes mellitus (T1DM) is a recently described rare occurrence following the administration of adjuvants such as coronavirus disease 2019 (COVID-19) vaccines. This systematic review aimed to review all available literature on the potential association between COVID-19 vaccines and T1DM.

**Methods:**

The Directory of Open Access Journals, MEDLINE, Google Scholar, and Scopus were systematically searched for all published studies from inception to July 2022. Articles reporting T1DM development within 8 weeks of administration of COVID-19 vaccine were included. Two reviewers independently performed the risk of bias assessment following the Joanna Briggs Institute (JBI) Critical Appraisal Checklist for Case Reports.

**Results:**

Eight eligible studies were retrieved, comprising 12 patients diagnosed with T1DM after being vaccinated with a COVID-19 vaccine. Six patients (50%) reported T1DM after receiving the second dose. Five patients (41.7%) presented with diabetic ketoacidosis, of which four presented within the first eight days after vaccination. Five patients (41.7%) had genetic susceptibility, with RNA binding motif protein 45 (*RBM45*/*DRB1*) and major histocompatibility complex, class II, DQ beta 1 (*HLA-DQB1*) mutations being prominent.

**Interpretation:**

In this review, we have shown a small number of new-onset diabetes cases coincidently occurring soon after the COVID-19 vaccine, especially in those with genetic susceptibility. Despite being older, these patients had a similar phenotype to T1DM. While there might be a causal relationship between COVID-19 vaccines and T1DM development, this should not influence decisions regarding vaccination since the overall benefit outweighs the risk. Further larger prospective trials are needed to assess causal relationship and to clarify the potential roles of COVID-19 vaccine-derived antigens in autoimmune disease development.

**Protocol registration:**

PROSPERO-CRD42022342093.

**Supplementary Information:**

The online version contains supplementary material available at 10.1186/s12902-023-01424-0.

## Significance statement

With the rapid and wide-spread rate of COVID-19 vaccines developed, many complications have appeared, including autoimmune diseases. In this review, we have shown a small number of new onset diabetes cases coincidently occurring soon after the COVID-19 vaccine. Despite being older, these patients will have a similar phenotype to type 1 diabetes and risk is higher in those with genetic susceptibility. Most will present with diabetic ketoacidosis and require insulin therapy. However, incidence remains rare, and the overall benefit outweighs the risk. Such a possible complication would be important to recognize to appropriately screen and treat affected patients.

**Supplementary Information:**

The online version contains supplementary material available at 10.1186/s12902-023-01424-0.

## Background

Since December 2019, the coronavirus disease 2019 (COVID-19) pandemic has imposed devastating health, social, and economic burdens on health systems worldwide [1, 2]. Many strategies and precautionary measures (e.g., social isolation, personal hygiene, wearing face masks, and frequent hand washing) have been implemented globally to limit COVID-19’s spread. However, a protective vaccine is required to achieve sufficient herd immunity to prevent the progression of the COVID-19 pandemic. Many vaccines have eventually been approved for clinical use, and around 12 billion doses have been administered globally [3, 4]. The most globally administered COVID-19 vaccines are based on messenger ribonucleic acids (e.g. mRNA-1273 [Moderna] and BNT162b2 [Pfizer-BioNTech]), viral vectors (e.g. ChAdOx1-S [Vaxzervria]), or inactivated viruses (e.g. CoronaVac [Sinovac Biotech]) [3, 4].

While the virus’ unprecedented toll caused the accelerated development of multiple vaccines by many biopharmaceutical companies, the breakneck pace of their development has raised safety concerns from the general population. A study on understanding the causes of COVID-19 vaccine hesitancy reported that concerns about vaccine side effects increased the odds of becoming vaccine hesitant by 31% [5]. Most of the reported adverse effects of COVID-19 vaccines are non-specific, including fever, fatigue, and headache [6]. However, emerging evidence indicates an intricate relationship between the COVID-19 vaccine and autoimmune/type 1 diabetes (T1DM) [7]. The administration of adjuvants such as COVID-19 vaccines, potentially containing virus-derived proteins, to genetically predisposed individuals could activate autoimmune cascades such as T1DM [7]. Despite vague evidence, the literature suggests additional autoimmune manifestations such as vaccine-induced thrombotic thrombocytopenia, autoimmune liver disease, immunoglobulin A (IgA) nephropathy, and Guillian–Barre syndrome [8]. In this context, T1DM is of great public concern. Therefore, this study aimed to review the literature and the evidence on the potential association between COVID-19 vaccines and T1DM. It describes the clinical presentation and outcomes of COVID-19-associated diabetes and assesses the quality of reports.

## Methods

### Protocol, registration, and search strategy

This systematic review of case reports was performed according to the Preferred Reporting Items for Systematic Reviews and Meta-Analysis (PRISMA) guidelines [9] and registered with the PROSPERO online database (identifier: CRD42022342093).

A systematic literature search was performed to identify all published studies from inception without language or country restrictions. The search was performed in July 2022 in the following databases: The Directory of Open Access Journal, MEDLINE, Google Scholar, and Scopus. It was conducted using the search terms: (‘COVID-19’ OR ‘SARS-CoV-2’) AND (‘Autoimmune Diseases’ OR ‘Type 1 Diabetes Mellitus’ OR ‘Autoimmune Disorder’ OR ‘Autoimmunity’) AND (‘Vaccines’ OR ‘COVID-19 Vaccines’ OR ‘Immunization’). Detailed queries are provided in Supplement Table 1. The reference lists of relevant articles were searched for additional studies in October 2022.

### Eligibility criteria

A search of case reports and case series of patients receiving a COVID-19 vaccine and developing T1DM within eight weeks of any vaccine dose was conducted. The T1DM diagnosis must have been established by either the presence of any positive antibodies known to cause the disease (anti-glutamic acid decarboxylase [anti-GAD] antibodies, anti-tyrosine phosphatase [anti-IA2] antibodies, islet cell antibodies, or insulin autoantibodies), or in the case of negative antibodies, and low C-peptide levels following the acute presentation. However, this study excluded articles with overlapping patient data, reporting diabetes development > 3 months after a vaccine injection, reporting cases where the diabetes diagnosis was insufficiently documented or unclear, and reporting cases known to have T1DM before vaccination.

### Study selection

**Fig. 1:**
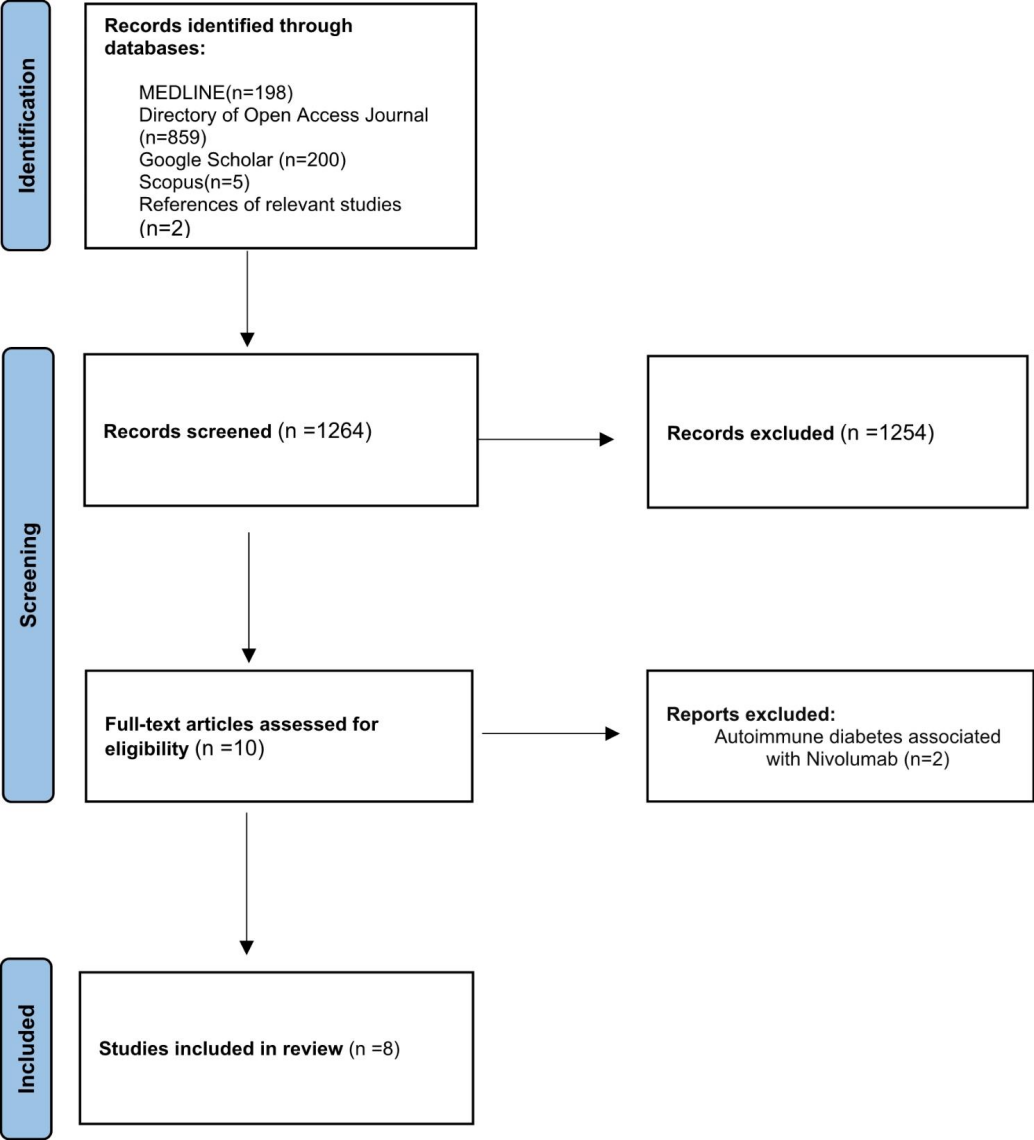
Study selection Flowchart

A detailed literature search was performed in the four databases mentioned above, identifying 1264 articles. Access to the predefined databases were granted by the Saudi Digital Library. All articles were exported to Microsoft Excel through which duplication removal and screening process were executed manually. Two authors (AsA and AbF) independently scrutinized all 1264 papers according to specific eligibility criteria. After review, 1254 articles were excluded, and ten were initially retained. An author raised conflicts about two articles, and a third author (SR) was involved in resolving the inter-author conflict, resulting in the exclusion of both articles. The remaining eight articles met the inclusion criteria and were included in the analysis (Fig. 1). The eligible studies were published between October 2021 and July 2022.

### Data extraction and quality assessment

Data were extracted in Microsoft Office Excel using a predefined template. The extracted data included the following information: author details, publication year, study characteristics, patient demographics, patients’ family and history of autoimmune diseases or diabetes mellitus, vaccine type, symptom onset (after which dose), signs and symptoms, diagnosis day after vaccination, diabetic ketoacidosis (DKA), antibody presence, fasting C-peptide levels, stimulated C-peptide levels, hemoglobin A1C (HbA1C) levels before vaccination, HbA1C levels at diagnosis, genetic susceptibility, and other T1DM triggers. Two reviewers independently performed the risk of bias assessment. Studies were scored as “yes,” “no,” or “not applicable,” following the Joanna Briggs Institute (JBI) Critical Appraisal Checklist for Case Reports based on an eight-item scale [10].

### Data analysis

Since this review is descriptive, we used descriptive statistics to describe the data with median (range) for continuous variables and frequencies and percentages for categorical variables. Microsoft Excel software was used for all calculations.

## Results

### Characteristics of the eligible studies and patients’ characteristics

This review included 12 cases from all eight included articles [11–18], summarized in Table 1. All patients were diagnosed with T1DM after being vaccinated with a COVID-19 vaccine. Seven of the 12 patients were female (58.3%). A summary of patients’ characteristics is provided in Table 2. Their median age was 49 years. Body mass index (BMI) was reported for seven patients, of which four were normal (18.5–24.9 kg/m^2^), two were underweight, and one was overweight. A family history of type 2 diabetes was recorded for three patients (25%). One patient (8.3%) had a family history of autoimmune diseases, including vitiligo and Hashimoto’s thyroiditis. Three patients (25%) had a personal history of other autoimmune diseases (vitiligo and Hashimoto’s thyroiditis). Moreover, two patients (16.7%) had a history of type 2 diabetes mellitus for > 7 years that was effectively controlled with diet modifications and oral antidiabetic agents. The Pfizer-BioNTech vaccine was given to seven patients (58.3%), the Moderna mRNA-1273 vaccine to two (16.7%), the CoronaVac to one (8.3%), and the ChAdox1-s vaccine to one (8.3%). The last patient (8.3%) received four doses of two different vaccines. They first received two CoronaVac doses, followed by two Pfizer-BioNTech doses, after which symptoms appeared.

Six patients (50%) reported T1DM after receiving their second dose, and symptoms appeared within eight weeks of administration. Additionally, five patients (41.7%) had T1DM after their first dose, and symptoms appeared within four weeks of administration. One patient (8.3%) had T1DM after their fourth dose, with symptoms appearing within three weeks. The shortest duration from vaccination to symptom onset was three days, while the longest was approximately eight weeks. DKA was recorded in five (41.7%) patients, one (8.3%) presented before developing DKA, while the rest (*n* = 6; 50%) had no available data on DKA at the time of T1DM presentation. C-peptide levels were quantified and documented for ten patients (83.3%), and antibodies were documented for nine (75%). Six of the 12 patients had their T1DM diagnosis established by low C-peptide levels and positive antibodies, three by low C-peptide levels only, and three by positive antibodies only. The most common antibody present was the anti-GAD (*n* = 8; 66.7%). Only five patients had valid data for HbA1c levels before vaccination; two had high values (HbA1c ≥ 7%), one had a prediabetic HbA1c value (HbA1c = 5.9%), and two had normal values (HbA1c ≤ 5.6%). Post-vaccine data showed that all patients had a very high HbA1c level indicative of T1DM, except one with no HbA1c data reported. The estimated mean HbA1c level at diagnosis was 9.96%. Triggers that might cause T1DM development including steroid use, pancreatic diseases, and viruses; have been reported nor established. Five patients (41.7%) had genetic susceptibility, with RNA binding motif protein 45 (*RBM45*/*DRB1*) and major histocompatibility complex, class II, DQ beta 1 (*HLA-DQB1*) mutations being prominent.

A basal-bolus insulin therapy regimen was used as the treatment mode in all but one patient to correct the acute presenting condition. The final patient refused insulin and was treated with diet modification, showing a good response. Eight of the 11 treated patients achieved sufficient glycemic control with insulin therapy only; the remaining three patients had missing data on their post-intervention status. Two of the seven patients who had data regarding their post-discharge condition stopped insulin and were being managed with diet modification only, showing good glycemic control (Supplementary Table 2).

The current study embodies systematic review of case reports, wherein the original articles have thoroughly complied with the ethical obligation of obtaining informed consent from the subjects under study.

**Table 1:** Clinical features and baseline laboratory results of the cases at presentation

	Case 1	Case 2	Case 3	Case 4	Case 5	Case 6	Case 7	Case 8	Case 9	Case 10	Case 11	Case 12
First Author (reference)	Yano et al. 2022 [11]	Bleve et al. 2022 [12]	Bleve et al. 2022 [12]	Sasaki et al. 2022 [13]	Sakurai et al. 2022 [14]	Sasaki et al. 2022 [15]	Tang et al. 2022 [16]	Patrizio et al. 2021 [17]	Aydoğan et al. 2022 [18]	Aydoğan et al. 2022 [18]	Aydoğan et al. 2022 [18]	Aydoğan et al. 2022 [18]
Country	Japan	Italy	Italy	Japan	Japan	Japan	China	Italy	Turkey	Turkey	Turkey	Turkey
Age (years)	51	57	61	73	36	45	50	52	56	48	27	36
Sex	F	F	F	F	F	F	M	M	M	M	F	M
BMI (kg/m^2^)	18.3	N/A	N/A	N/A	N/A	20.6	18.1	N/A	27.4	21.9	20	22.8
Medical history	None	N/A	Acquired hypothyroidism	- Osteoporsis - Non-tuberculous mycobacterial infection - Diet-controlled type 2 diabetes	None	Bronchial asthma	None	- Vitiligo - T2DM for 8 years on oral agents	Vitiligo and Hashimoto thyroiditis	None	None	None
Family history	T2DM (father)	T2DM, vitiligo, Hashimoto’s thyroiditis	N/A	None	None	N/A	T2DM (mother)	N/A	N/A	None	None	None
Type of vaccination	Moderna mRNA-1273	ChAdOx1-S	Pfizer-BioNTech	Moderna mRNA-1273	Pfizer-BioNTech	Pfizer-BioNTech	CoronaVac	Pfizer-BioNTech	Pfizer-BioNTec	Pfizer-BioNTec	Pfizer-BioNTec	Pfizer-BioNTec and CoronaVa
Duration from vaccination to symptoms onset (weeks)	4 (28 days)	1 (8 days)	Since receiving the 2nd dose of the vaccine	4	< 1 (3 days)	1 (6 days)	1 (6 days)	4	2 (15 days)	8	3	3
Dose after which the symptoms appeared	1st	1^s^	2nd	2nd	1^s^	1^s^	1^s^	2nd	2nd	2nd	2nd	4th
DKA at presentation	Yes	N/A	N/A	N/A	Yes	Yes	Yes	N/A	No	N/A	N/A	Yes
Triggers (e.g. steroids use, pancreatic disease, viruses)	No infection	No infection	N/A	N/A	No infection	No infection	N/A	N/A	N/A	N/A	N/A	N/A
**Laboratory findings**												
HbA1c (%) before vaccination (reference range)	5.6 (4.6–6.2)	N/A	N/A	> 7%	N/A	N/A	N/A	7%	5.9 (4.0-5.6)	5.6 (4.0-5.6)	N/A	N/A
HbA1c (%) at diagnosis (reference range)	10.3 (4.6–6.2)	10.4	11.5	9.3 (4.9–5.9)	7 (4.6–6.1)	7.6 (4.6–6.2)	“Near normal”	10.1% (4.6–6.1)	8.2 (4.0-5.6)	10.1 (4.0-5.6)	12.5 (4.0-5.6)	12.6 (4.0-5.6)
Anti-GAD antibodies presence	No	Yes	Yes	Yes	No	No	No	Yes	Yes	Yes	Yes	Yes
Other antibodies presence	- Insulin autoantibodies - TPOAb*	- Anti-IA2 - Anti-TransGlut IgA	TPOAb	Insulin autoantibodies	None	None	None	- TRAb^ - TgAb - TPOAb	N/A	N/A	N/A	N/A
C-peptide (ng/mL) (refrence range)	0.4 (0.80–2.50)	N/A	N/A	0.54 (0.74–3.18)	0.35 (0.8–2.3)	0.33 (0.80–2.5)	Undetectable/Low levels	1.0 (1.0-3.5)	1.5 (1.1–4.4)	0.97 (1.1–4.4)	0.87 (1.1–4.4)	0.39 (1.1–4.4)
HLA-DNA typing (genetic susceptibility)	DRB1*09:01-DQB1*03:03	N/A	N/A	DRB1 *04:05:01-DQB1*04:01:01	DRB1*0405-DQB1*0401	DRB1*04:05:01/ *13:02:01 and DQB1*04:01:01/*06/04:01	DQB1*02:03/ 03:03 and DRB1*09:01/09:01	N/A	N/A	N/A	N/A	N/A

Abbreviations: T2DM: Type 2 diabetes mellitus, Anti-GAD: anti-glutamic acid decarboxylase antibodies, Anti-IA2: anti-tyrosine phosphatase antibodies. Anti-TransGlut IgA: anti- transglutaminase IgA antibody, TPOAb: thyroid peroxidase antibodies, TRAb: thyrotropin receptor antibodies, TgAb: thyroglobulin antibodies, DKA: diabetic ketoacidosis, N/A: information not available, F: Female, M: Male. *This patient had normal levels of thyroid hormones; thus, diagnosed with latent autoimmune thyroid disease. ^This patient did not only develop T1DM after COVID-19 vaccination, but also developed Graves’ disease

**Table 2:** Summary of patients’ characteristics

Variable		Descriptive statistics
Age, *median (range)* years		50.5 (27–73)
Sex, *n (%)*	Female	7 (58.3)
	Male	5 (41.7)
Type of vaccine received, *n (%)*	Moderna mRNA-1273	2 (16.7)
	ChAdOx1-S	1 (8.3)
	Pfizer-BioNTec	7 (58.3)
	CoronaVac	1 (8.3)
	Pfizer-BioNTec and CoronaVa	1 (8.3)
Duration from vaccination to symptoms onset, *median (range)* weeks		2.5 (0–8)
Dose after which symptoms appeared, *n (%)*	1st dose	5 (41.7)
	2nd dose	6 (50)
	4th dose	1 (8.3)
Past medical history of autoimmune disease, *n (%)*		3 (25)
Diabetic ketoacidosis at presentation (DKA), *n (%)*		5 (41.7)

### Quality assessment

The overall quality of the included studies was intermediate to good. Two studies were classified as being of intermediate quality [12, 17], while the remaining studies as good quality (Table 3). Question #7 from the JBI tool was not applicable to this systematic review because no intervention was studied. Details of each study’s quality assessment are presented in the Appendix (Supplementary Table 3).

**Table 3:** Characteristics of articles included

First author [reference number]	Quality score*	Overall quality
Yano et al. 2022 [11]	6	Good
Bleve et al. 2022 [12]	4	Intermediate
Sasaki et al. 2022 [13]	7	Good
Sakurai et al. 2022 [14]	6	Good
Sasaki et al. 2022 [15]	6	Good
Tang et al. 2022 [16]	7	Good
Patrizio et al. 2021 [17]	4	Intermediate
Aydoğan et al. 2022 [18]	7	Good

*JBI risk assessment tool was used

## Interpretation

To our knowledge, this is the first and largest systematic review of reported cases of COVID-19 vaccine-associated T1DM. This systematic review of eight articles identified 12 case reports with T1DM, defined by either the presence of autoantibodies or low C-peptide levels at diagnosis. Four different vaccines were administered to the 12 patients. They were all mRNA, recombinant DNA, viral vector, or inactivated virus vaccines, with most cases (83.3%) given an mRNA-based vaccine. Of the 12 patients, 41.7% developed T1DM symptoms after their first dose, of which 80% presented with symptoms within the first 10 days. However, 50% developed symptoms after their second dose. The average time to symptom onset varied from days to weeks, with the shortest duration being three days and the longest being eight weeks after vaccination. Five patients (41.7%) presented with DKA, an uncontrolled diabetes complication, of which four presented within the first eight days after vaccination.

Upon assessment of the patients in this study, it became evident that aside from genetic susceptibility and a history of autoimmune diseases, there were no risk factors for developing T1DM. There were no triggers for developing autoimmune diseases, such as infections, steroid use, or pancreatic diseases. BMIs were mostly normal or underweight. All patients were far from T1DM’s peak incidence age, usually within childhood (Table 4). Genetic susceptibility was only examined for five patients. One patient had a family history of autoimmune diseases. Three patients were already diagnosed with autoimmune diseases, such as Hashimoto’s thyroiditis and vitiligo. While it is likely for those with autoimmune diseases to develop other autoimmune diseases, a trigger is usually required. Two patients with elevated HbA1c levels before vaccination were known to have type 2 diabetes. However, their HbA1c level increased significantly after vaccination, and they likely converted into T1DM triggered by the vaccine with autoantibody development. Although long-standing type 2 diabetes can lead to beta cell failure and low C-peptide, the fact that their diabetes was well controlled on oral agents, and they developed autoantibodies makes them less likely to be type 2 diabetes. Two patients had no data of C-peptide presented with a significantly elevated HgA1c (> 10%). However, both had either a personal or family history of autoimmune disease and were not diabetics before. In addition to the significant elevation in HgA1C (> 10%), the presence of type 1 diabetes-related antibodies might support the hypothesis that these 2 cases are type 1 rather than type 2 diabetes. Furthermore, three patients presented with a rapid development of DKA within one week of receiving COVID-19 vaccines. Although this might be a coincidence, the fact that their HbA1C levels was around 7% at time of diagnosis indicates that their severe hyperglycemia was of very recent onset. Moreover, these patients had genetic susceptibility and these genes have been linked to fulminant type 1 diabetes, which might be the reason for this very quick development of ketonemia.

**Table 4:** Characteristics similarities & differences between types of autoimmune diabetes [19, 20]

Characteristics	Usual Type 1 Diabetes Mellitus	Late Autoimmune Diabetes in Adults (LADA)	Autoimmune Diabetes following COVID-19 Vaccine
Age at onset	Most commonly in Childhood	Adult age (30–50 years of age); but may occur at any age	Adult age (25–75 years of age)
Typical weight	Normal – underweight	Normal – overweight	Normal – underweight
Symptoms at onset	Acute	Insidious	Acute
Time to requiring insulin	At onset	Months-years	At onset
Presence of Autoantibodies	Yes	Yes	Yes
C-peptide levels	Low-undetectable	Low-normal	Low-undetectable
Personal or family history of other autoimmune disease	Yes	Yes	Yes
Genetic susceptibility	Yes	Yes	Yes
DKA at presentation	Common	Rare	Common
Honeymoon phase	Yes (usually after 6–18 months)	No	Possible (after 4–8 weeks)

Following T1DM presentation and diagnosis, all but one patient (*n* = 11) were managed with an intensive basal-bolus insulin therapy regimen. Two patients steadily decreased their insulin doses and discontinued insulin treatment due to recurrent hypoglycemic episodes. Those who discontinued insulin therapy were proposed to have entered the honeymoon phase (or partial remission). This is a phase within some diabetic patients’ courses involving increased pancreatic B-cell activity and insulin sensitivity, resulting in a progressive decrease in insulin dependence to the point where they no longer require insulin therapy [8]. This phase was reported to occur in 3–61% of newly diagnosed T1DM patients, with the highest incidence within the first six months to one year after diagnosis, but its pathophysiology remains unclear [8]. Many patients with partial remission require low-dose insulin therapy (< 0.5 U/kg/day) or some oral antidiabetic medications. Those who completely refrain from medication use may be categorized as having complete remission, provided they maintain HbA1c levels < 6% and normal glycemic levels without any medications [8, 21, 22]. Interestingly, two of the four patients with post-discharge follow-up data (50%) were off insulin and showed good glycemic control, indicating a possible honeymoon phase. This transition occurred around three months after diagnosis, which is relatively shorter than in usual T1DM patients. The lack of long-term follow-up information for most of the included cases precludes us from reaching a definitive conclusion regarding the rate and duration of remission in such cases.

COVID-19 vaccines have been proposed to act as triggers for autoimmune diseases via multiple pathways, including immune system hyperstimulation, autoantibody formation, and molecular mimicry [23]. The severe acute respiratory syndrome coronavirus 2 (SARS-COV-2) vaccine has been suggested to cause a “cytokine storm” involving the release of multiple cytokines to induce an inflammatory state in those with mild to severe infections. Hyperstimulation is associated with autoimmune disease development since those with severe COVID-19 developed anti-phospholipid and anti-nuclear antibodies, interferon-neutralizing autoantibodies, and antineutrophil cytoplasmic antibodies against myeloperoxidase (MPO/p-ANCA) and proteinase 3 (PRTN3/c-ANCA) [4].

Furthermore, T1DM is not the only autoimmune disease associated with COVID-19. Multiple case reports have suggested a relationship between the SARS-CoV-2 vaccine and Guillain–Barre syndrome [24], Graves’ disease [6, 25], warm and cold autoimmune hemolytic anemia [26], and Kawasaki disease [27]. The basis for developing various autoimmunity forms following COVID-19 vaccination is thought to sequence homology between SARS-CoV-2 and human proteins. New antibodies created to attack the virus have the potential to cross-react with the host’s cells. Many human proteins shared with SARS-COV-2 have been identified as potentially pathogenic when perturbed (e.g. mutation, alteration, and improper function) [23].

As indicated by autoimmunity development, mRNA vaccines result in more post-vaccination T1DM cases than all other vaccine types. The mRNA-based COVID-19 vaccines have a higher pooled risk ratio for developing adverse events and local adverse reactions following immunization than all other vaccine types [6]. Furthermore, while rare, developing autoimmunity following vaccination is a known risk and is categorized under the term autoimmune/inflammatory syndrome by adjuvants (ASIA syndrome), covering vaccination-induced side effects and other complications. This syndrome is thought to develop in those with pre-existing genetic risk factors after exposure to an adjuvant in the vaccine by activating autoimmune pathways.

Though not explicitly explained, higher adverse reaction rates following mRNA vaccination are important since they greatly influence vaccine hesitancy and compliance in populations. Sallam’s [28] systematic review of vaccine acceptance rates showed a large variance in SARS-CoV-2 vaccine acceptance among countries, ranging from as low as 23.4% and 28.3% in Kuwait and Jordan, respectively, to as high as 97.0% and 94.3% in Ecuador and Malaysia, respectively. The most common causes of hesitancy were low disease risk perception, lack of trust in vaccination safety and effectiveness, and vaccine affordability and delivery [28]. Therefore, when presenting information like that offered by this study, it is important to place the findings in the context of the entire vaccination program. Over 12.85 billion doses of COVID-19 vaccines [29] have been administered, with a minority of individuals experiencing severe adverse effects due to vaccination. The benefit of vaccinating the population and preventing disease propagation far outweighs the individual vaccination risks, as shown by the 6.6 million deaths worldwide [29] and severe hospitalizations that strained healthcare systems globally, resulting in the inability to provide care to many individuals.

### Limitations

Our review had multiple limitations. First, given that included articles were case reports and series, missing information is a significant issue. For example, multiple parameters were expected to act as baseline measurements for the participants in this study, including BMI, HbA1c level, genetic susceptibility, and a history of exposure to autoimmune disease triggers such as infection. They are used to assess whether the phenotype of vaccine-induced diabetes is similar to T1DM. Unfortunately, many of these measurements were missing in the assessed case reports. In two of the case reports an intermediate overall quality score was seen. This low score was attributed to important missing variables in terms of diagnosis and laboratory work up. Despite the lack of major risk factors for developing T1DM in patients with complete data, these sets of information or their lack thereof limit our ability to conclude with certainty that vaccination is the cause of T1DM development. Secondly, there might be an underdiagnosis and underappreciation of T1DM development triggered by COVID-19 vaccination, especially in low-income countries where testing for antibodies and C-peptides might be limited. In such countries, most of these patients might be labelled as type 2 diabetes, given their older age at diagnosis.

### Conclusions

We have shown in this review a small number of new onset diabetes cases coincidently happening soon after COVID-19 vaccine, especially in those with genetic susceptibility. Despite being older, these patients had a similar phenotype to T1DM. Most presented with DKA and required insulin therapy but needed to be monitored closely since their insulin requirement might rapidly decline. Moreover, sudden unexplained severe hyperglycemia and increased HbA1c with development of diabetes-related autoantibodies in otherwise well-controlled type 2 diabetes should alert physicians about the possibility of conversion to T1DM. There might be an underappreciation and underdiagnosing of this COVID-19-induced T1DM. Nevertheless, its incidence remains rare compared to the huge number of vaccine doses given worldwide. While there might be an association in a few case reports between COVID-19 vaccines and T1DM development, this should not influence decisions regarding vaccination since the overall benefit outweighs the risk. Further larger prospective trials are needed to assess causal relationship and to clarify the potential roles of COVID-19 vaccine-derived antigens in autoimmune disease development.

### Electronic supplementary material

Below is the link to the electronic supplementary material.

Supplementary Material 1

## Data Availability

The details of extracted data, and quality assessment are in the appendices. Further data may be available upon a reasonable request to the corresponding author.

## Declarations

### Approval date of registry and the registration no. of the study/trial

Protocol registration: PROSPERO-CRD42022342093, Date: 04/07/2022.

## References

[1] Pak A, Adegboye OA, Adekunle AI, Rahman KM, McBryde ES, Eisen DP (2020). Economic Consequences of the COVID-19 outbreak: the need for epidemic preparedness. Front Public Heal.

[2] Chang AY, Cullen MR, Harrington RA, Barry M (2021). The impact of novel coronavirus COVID-19 on noncommunicable disease patients and health systems: a review. J Intern Med.

[3] Creech CB, Walker SC, Samuels RJ (2021). SARS-CoV-2 vaccines. JAMA.

[4] Mathieu E, Ritchie H, Rodés-Guirao L, Appel C, Giattino C, Hasell J et al. Coronavirus Pandemic (COVID-19). Our World in Data. 2020. https://ourworldindata.org/coronavirus. Accessed 26 Oct 2022.

[5] Hassen HD, Welde M, Menebo MM (2022). Understanding determinants of COVID-19 vaccine hesitancy; an emphasis on the role of religious affiliation and individual’s reliance on traditional remedy. BMC Public Health.

[6] Kouhpayeh H, Ansari H (2022). Adverse events following COVID-19 vaccination: a systematic review and meta-analysis. Int Immunopharmacol.

[7] Bragazzi NL, Hejly A, Watad A, Adawi M, Amital H, Shoenfeld Y (2020). ASIA syndrome and endocrine autoimmune disorders. Best Pract Res Clin Endocrinol Metab.

[8] Moole H, Moole V, Mamidipalli A, Dharmapuri S, Boddireddy R, Taneja D (2015). Spontaneous complete remission of type 1 diabetes mellitus in an adult – review and case report. J Community Hosp Intern Med Perspect.

[9] Page MJ, McKenzie JE, Bossuyt PM, Boutron I, Hoffmann TC, Mulrow CD (2021). The PRISMA 2020 statement: an updated guideline for reporting systematic reviews. BMJ.

[10] Critical Appraisal Tools | JBI. https://jbi.global/critical-appraisal-tools. Accessed 20 Nov 2022.

[11] Yano M, Morioka T, Natsuki Y, Sasaki K, Kakutani Y, Ochi A (2022). New-onset type 1 diabetes after COVID-19 mRNA vaccination. Intern Med.

[12] Bleve E, Venditti V, Lenzi A, Morano S, Filardi T (2022). COVID-19 vaccine and autoimmune diabetes in adults: report of two cases. J Endocrinol Invest.

[13] Sasaki H, Itoh A, Watanabe Y, Nakajima Y, Saisho Y, Irie J (2022). Newly developed type 1 diabetes after coronavirus disease 2019 vaccination: a case report. J Diabetes Investig.

[14] Sakurai K, Narita D, Saito N, Ueno T, Sato R, Niitsuma S (2022). Type 1 diabetes mellitus following COVID-19 RNA-based vaccine. J Diabetes Investig.

[15] Sasaki K, Morioka T, Okada N, Natsuki Y, Kakutani Y, Ochi A (2022). New-onset fulminant type 1 diabetes after severe acute respiratory syndrome coronavirus 2 vaccination: a case report. J Diabetes Investig.

[16] Tang X, He B, Liu Z, Zhou Z, Li X. Fulminant type 1 diabetes after COVID-19 vaccination. Diabetes Metab. 2022;48. 10.1016/J.DIABET.2022.101324. 10.1016/j.diabet.2022.101324 PMC8786596 35091092

[17] Patrizio A, Ferrari SM, Antonelli A, Fallahi P. A case of Graves’ disease and type 1 diabetes mellitus following SARS-CoV-2 vaccination. J Autoimmun. 2021;125. 10.1016/J.JAUT.2021.102738. 10.1016/j.jaut.2021.102738 PMC8506108 34653776

[18] Aydoğan B, Ünlütürk U, Cesur M (2022). Type 1 diabetes mellitus following SARS-CoV-2 mRNA vaccination. Endocrine.

[19] Association AD (2017). 2. Classification and diagnosis of diabetes: Standards of Medical Care in Diabetes—2018. Diabetes Care.

[20] Grill VLADA (2019). A type of diabetes in its own right?. Curr Diabetes Rev.

[21] Infante M, Fabbri A, Padilla N, Pacifici F, Di Perna P, Vitiello L (2022). BNT162b2 mRNA COVID-19 vaccine does not impact the Honeymoon Phase in Type 1 diabetes: a Case Report. Vaccines.

[22] Oyibo SO. Partial remission of diabetes in a young adult while testing positive for several islet cell autoantibodies: a Case Report, Literature Review, and patient perspective. Cureus. 2022;14. 10.7759/cureus.25746 PMC9177224 35702638

[23] Dotan A, Muller S, Kanduc D, David P, Halpert G, Shoenfeld Y (2021). The SARS-CoV-2 as an instrumental trigger of autoimmunity. Autoimmun Rev.

[24] Rahimi K (2020). Guillain-Barre syndrome during COVID-19 pandemic: an overview of the reports. Neurol Sci.

[25] Lui DTW, Lee KK, Lee CH, Lee ACH, Hung IFN, Tan KCB (2021). Development of Graves’ Disease after SARS-CoV-2 mRNA vaccination: a Case Report and Literature Review. Front Public Heal.

[26] Lazarian G, Quinquenel A, Bellal M, Siavellis J, Jacquy C, Re D (2020). Autoimmune haemolytic anaemia associated with COVID-19 infection. Br J Haematol.

[27] Jones VG, Mills M, Suarez D, Hogan CA, Yeh D, Bradley Segal J, et al. COVID-19 and Kawasaki Disease: Novel Virus and Novel Case. Hosp Pediatr. 2020;10. 10.1542/HPEDS.2020-0123. 10.1542/hpeds.2020-0123 32265235

[28] Sallam M (2021). COVID-19 Vaccine Hesitancy Worldwide: a concise systematic review of Vaccine Acceptance Rates. Vaccines.

[29] COVID-19 Map - Johns Hopkins Coronavirus Resource Center. https://coronavirus.jhu.edu/map.html. Accessed 22 Nov 2022.

