# Supplementary material for: Autoimmune diabetes mellitus after COVID-19 vaccination in adult population: a systematic review of case reports
Source: BMC Endocr Disord. 2023 Aug 4;23:164. doi: 10.1186/s12902-023-01424-0 (PMC10403898; doi:10.1186/s12902-023-01424-0)
Supplement: Supplementary file 1 — Supplementary Material 1 [file 12902_2023_1424_MOESM1_ESM.docx]

**Supplementary Material**

**Supplementary Table 1.** Search strategy: Detailed queries

| **Search engine** | **Database** | **Query** | **Number of articles found (Results from July 2022)** |
| --- | --- | --- | --- |
| Saudi digital library | MEDLINE | (‘COVID-19’ OR ‘SARS-CoV-2’) AND (‘Autoimmune Diseases’ OR ‘Type 1 Diabetes Mellitus’ OR ‘Autoimmune Disorder’ OR ‘Autoimmunity’) AND (‘Vaccines’ OR ‘COVID-19 Vaccines’ OR ‘Immunization’) | 198 |
|  | Scopus |  | 5 |
|  | The Directory of Open Access Journal |  | 859 |
| Google scholar |  |  | 15,900  (From which we considered the first 10 pages “most relevant topics” accounting for 200 articles) |

**Supplementary Table 2.** Signs, symptoms**,** and management,

| **First author (reference)** | **Signs and symptoms** | **Management** | **Response** |
| --- | --- | --- | --- |
| Yano et al. 2022 (11) | **After 28 days of the first dose, and got worse after the second dose:**  - Thirst  - Fatigue  - Polyuria  - Polydipsia  - Increased intake of soda  - Weight loss (3kg)  - Hyperglycemia  - Tachycardia  - Mild dehydration | 4.0 L of intravenous saline  Intravenous infusion of regular insulin  started at 0.1 unit/kg/h and then was adjusted afterwards. Eventually, patient complied to subcutaneous insulin therapy:   - Insulin Glargine U-300 (14 units) before supper - Insulin lispro before meals (6 units, 4 units, and 6 units before breakfast, lunch, and supper, respectively) | Patient’s acute condition has improved, and diabetic ketoacidosis has completely resolved. Patient has achieved adequate glycemic control with subcutaneous insulin therapy. However, no information available on post-discharge glycemic control. |
| Bleve et al. 2022 (12) | **After 8 days of the first dose:**  - Polydipsia  - Hyperglycemia  - Glycosuria  - Ketonuria  - Asthenia | 1^st^ Patient: 0.9% NaCl solution and rapid insulin administration to correct the acute condition. Basal-bolus insulin therapy regimen was initiated upon discharge. | Hyperglycemic state was corrected. No other information |
|  | **Since administration of the second dose:**  - Polyuria  - Polydipsia  - Asthenia  **After 26 days of receiving the second dose:**  - Severe dyspnea  - Nausea  - Abdominal pain  - Metabolic acidosis | 2^nd^ Patient: 0.9% NaCl solution and rapid insulin administration to correct the acute condition. Basal-bolus insulin therapy regimen was initiated upon discharge. | Hyperglycemic state was corrected. No other information |
| Sasaki et al. 2022 (13) | **Four weeks after 2^nd^ dose:**  - Anorexia  - Hyperglycemia  - Fatigue  - Nausea and vomiting | Initiation of intensive insulin therapy | Patient has achieved adequate glycemic control. Blood test done 12 weeks after her 2nd vaccine dose showed that he is still insulin-dependent with low C-peptide. |
| Sakura et al. 2022 (14) | **After 3 days from the first dose:**  - Thirst  - Polydipsia  - Polyuria  - Palpitations  - Loss of appetite  - Fatigue | Intravenous fluids and rapid insulin infusion to correct the acute condition. Afterwards, started on subcutaneous multiple injections of insulin during the admission. | Patient’s acute condition has been stabilized. After the subcutaneous insulin therapy, patient was discharged in a stable condition. No information available on post-discharge glycemic control. |
| Sasaki et al. 2022 (15) | **After 6 days from the first dose:**  - Nausea  - Abdominal pain  - Hyperglycemia  - Weight loss (6kg)  - Metabolic acidosis | Intensive insulin therapy with Basal-bolus insulin therapy regimen was promptly initiated to correct the acute condition.  patient complied to subcutaneous insulin therapy:   - Insulin Glargine U-300 (14 units) before supper - Insulin lispro before meals (8 units, 10 units, and 8 units breakfast, lunch, and supper, respectively) | Patient’s acute condition has improved, and diabetic ketoacidosis has completely resolved. Patient has achieved adequate glycemic control with subcutaneous insulin therapy. No information available on post-discharge glycemic control. |
| Tang et al. 2022 (16) | **Five days after receiving the first dose:**  - Fever  - Polydipsia  - Polyuria  - Hyperglycemia  - Diabetic ketoacidosis | Intravenous fluids and insulin infusion for the acute condition. Afterwards, subcutaneous insulin therapy was initiated to maintain the glycemic control | Two weeks after the acute presentation, serum ketone bodies became negative but slightly elevated HbA1c and low serum C-peptide levels. Four weeks after acute presentation, he was still insulin-dependent with low serum C-peptide levels. |
| Patrizio et al. 2021 (17) | **Four weeks after 2^nd^ dose:**  - Night fever  - Weight loss (7kg)  - Asthenia  - Thyromegaly  - Overt hyperthyroidism  - Graves features on ultrasound | Insulin therapy was initiated | N/A |
| Aydoğan et al. 2022 (18) | **Fifteen days after 2^nd^ dose:**  - Fatigue  - Dry mouth  - Polyuria  - Wight loss (5kg in a week) | 1^st^ Patient: Rapid insulin administration to correct the acute condition. Then Basal-bolus insulin therapy regimen was initiated. | Patient’s acute condition has improved. During 3-months period follow up, patient insulin requirements were  steadily reduced and discontinued insulin treatment at the 3-month visit due to recurrent hypoglycemic episodes. (Honeymoon phase) |
|  | **Eight weeks after 2^nd^ dose:**  - Fatigue  - Hyperglycemia | 2^nd^ Patient: Patient refused to initiate insulin treatment but adopted  low-carbohydrate diet that resulted in frequent hypoglycemic events in continuous  glucose monitoring. | Frequent hypoglycemic episodes in continuous glucose monitoring with low-carbohydrate diet |
|  | **Three weeks after 2^nd^ dose:**  - Blurred vision  - Polyuria  - Polydipsia  - Weight loss  - Vaginal candidiasis | 3^rd^ Patient: Medical nutrition therapy and a basal-bolus insulin therapy regimen was initiated. Her insulin requirements were  gradually reduced, and she discontinued insulin treatment. | During follow up, patient insulin requirements were steadily reduced, and she discontinued insulin treatment. |
|  | **Three weeks after 4^th^ dose:**  - Fatigue  - Dizziness  - Unintentional weight loss  - Decreased skin turgor  - Dry mouth | 4^th^ Patient: Intravenous fluids and rapid insulin administration to correct the acute condition. Basal-bolus insulin therapy regimen was initiated upon discharge. | Patient’s acute condition has improved. At the 2^nd^ month follow up, basal bolus insulin dose was reduced. |

**Supplementary Table 3.** Risk of bias assessment.

| **Authors (reference)** | **Q1** | **Q2** | **Q3** | **Q4** | **Q5** | **Q6** | **Q7** | **Q8** |
| --- | --- | --- | --- | --- | --- | --- | --- | --- |
| Yano et al. 2022 (11) | Yes | Yes | Yes | Yes | Yes | No | N/A | Yes |
| Bleve et al. 2022 (12) | Yes | No | Yes | Yes | Yes | No | N/A | No |
| Sasaki et al. 2022 (13) | Yes | Yes | Yes | Yes | Yes | Yes | N/A | Yes |
| Sakura et al. 2022 (14) | Yes | Yes | Yes | Yes | Yes | No | N/A | Yes |
| Sasaki et al. 2022 (15) | Yes | Yes | Yes | Yes | Yes | No | N/A | Yes |
| Tang et al. 2022 (16) | Yes | Yes | Yes | Yes | Yes | Yes | N/A | Yes |
| Patrizio et al. 2021 (17) | Yes | Yes | No | Yes | Yes | No | N/A | No |
| Aydoğan et al. 2022 (18) | Yes | Yes | Yes | Yes | Yes | Yes | N/A | Yes |

1. Were patient’s demographic characteristics clearly?
2. Was the patient’s history clearly described and presented as a timeline?
3. Was the current clinical condition of the patient on presentation clearly described?
4. Were diagnostic tests or assessment methods and the results clearly described?
5. Was the intervention(s) or treatment procedure(s) clearly described?
6. Was the post-intervention clinical condition clearly described?
7. Were adverse events (harms) or unanticipated events identified and described?
8. Does the case report provide takeaway lessons?
